# Underlying Ehlers-Danlos syndrome discovered during neuro-ophthalmic evaluation of concussion patients: a case series

**DOI:** 10.1186/s12886-019-1174-2

**Published:** 2019-07-25

**Authors:** Abhishek Gami, Eric L. Singman

**Affiliations:** 1 Johns Hopkins School of Medicine, Baltimore, USA; 2 Wilmer Eye Institute, Johns Hopkins Hospital, Baltimore, USA

**Keywords:** Concussion, Ehlers-Danlos syndrome, Rehabilitation, Convergence insufficiency, Traumatic brain injury

## Abstract

**Background:**

The Ehlers-Danlos syndromes are a heterogenous group of diseases that cause connective tissue defects. At present, there are no published reports focusing upon the neuro-ophthalmic symptoms that might occur in EDS patients after mild traumatic brain injury. The demographics and clinical course of seven patients with subclinical EDS and mild traumatic brain injury are presented.

**Case presentation:**

This series describes patients with Ehlers Danlos Syndrome whose diagnosis was discovered in a neuro-ophthalmic clinic for brain injury. Patient demographics and neuro-ophthalmological symptoms are presented.

**Conclusions:**

Patients with subclinical EDS and brain injury may experience a slower, less complete recovery course. Heightened awareness of undiagnosed or underlying Ehlers Danlos Syndrome is important for patients and providers.

## Background

The Ehlers-Danlos syndromes (EDSs) are a group of heritable disorders that cause connective tissue defects. Signs of EDSs include hypermobile joints, loose skin, and fragile tissues due to defective connective tissue synthesis [1]. Thirteen types of EDS have been identified with significant phenotypic heterogeneity. The overall prevalence of EDSs is between 1 in 2500 to 1 in 5000, and the classic and hypermobile EDS variants are the most common phenotype [2]. It is likely that the milder forms may be underdiagnosed, due to their phenotypic heterogeneity. When hypermobile EDS is suspected, a detailed history should be obtained to assess hypermobility, easy bruising or bleeding, poor wound healing, gastrointestinal issues, repeated or voluntary joint dislocations, history of hernia or muscle injury in the setting of minor trauma so that the clinical diagnosis can be made [1]. However, studies and anecdotal observation have suggested that subclinical EDS can also be unmasked by a physical trauma event due to the tissue fragility associated with EDS [3]. Neurological and spinal manifestations of EDS that can become manifest in these patients can include cervicogenic headache, migraine, cerebrospinal fluid hyper- or hypotension, Chiari malformation, and atlantoaxial/craniocervical instability [4]. At present, there are no published reports focusing upon the neuro-ophthalmic symptoms that might occur in EDS patients after mild traumatic brain injury (TBI). In the following case series, we report neuro-ophthalmic manifestations that developed in patients with underlying EDS or joint hypermobility disorders following TBI. All patients in this study were provided a thorough ophthalmic examination by a single provider (ELS) as well as complete neuro-ophthalmic evaluation for visuomotor concerns commonly associated with concussion. In addition, all patients ultimately were confirmed to have hypermobile EDS, i.e., that type of EDS for which the specific genetic mutation in unknown, by geneticist-physicians evaluating the patients after that diagnosis was suggested in the TBI clinic. Written consent for using de-identified information was obtained by all patients and this study was approved by the Johns Hopkins Hospital Institutional Review Board (IRB00168974). The patient data can be accessed by contacting the corresponding author.

## Case presentations

### Patient 1

**Fig. 1:**
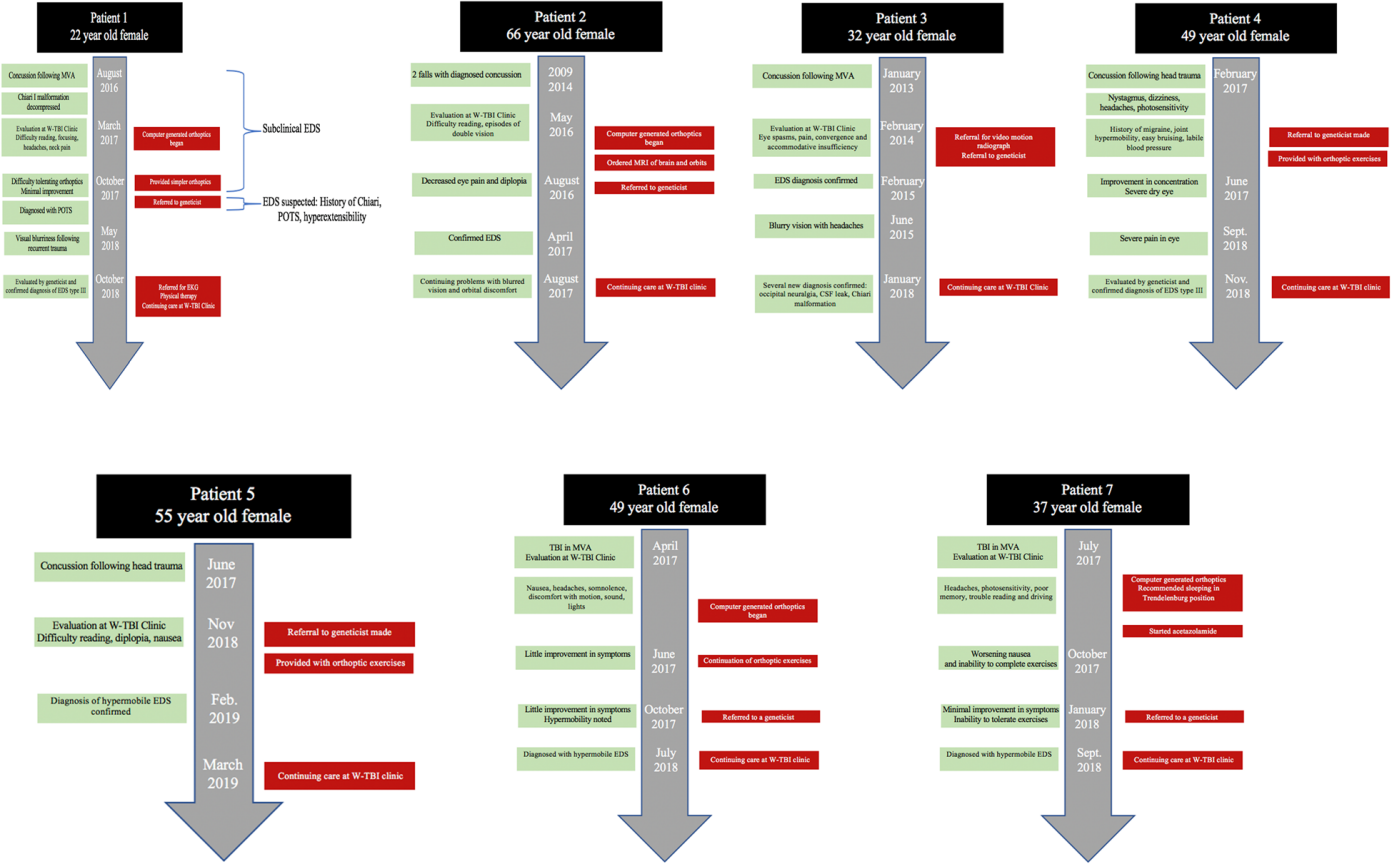
Patient Timelines for each of the seven patients in this case series. Each timeline provides the patient number, gender and age (black box), health events such as inciting trauma, evaluations, surgeries, etc. (green boxes), recommendations/orders offered in the W-TBI clinic (red boxes), month and year (vertical grey arrow)

Patient 1 is a 22-year-old woman who suffered a concussion with no loss of consciousness in August 2016 from a motor vehicle accident. A previously subclinical Chiari I malformation that had become symptomatic following the accident was discovered and was decompressed in November 2016 but her symptoms continued and even worsened. In March 2017, she was evaluated at the W-TBI clinic, reporting difficulties with reading and concentrating, as well as visual tracking and focusing. Review of systems elicited complaints of headaches, neck and back pain, balance problems, fatigue, heartburn, frequent urination, swallowing problems, and impaired cognition, dysphagia and choking. Patient 1 was found to have convergence insufficiency as well as deficiency of smooth pursuits upon examination (as evidenced by nausea and dizziness while attempting to track a pendulum). Computer-generated orthoptic exercises for home use were provided to the patient with instructions to return in three months for re-evaluation. In June 2017, Patient 1 returned to the clinic after completing her orthoptic exercises but was still experiencing trouble reading and concentrating. Review of systems elicited further complaints of headache, neck and back pain, dizziness and poor balance. She mentioned that the orthoptic exercises caused her to become nauseous and develop headaches. Patient 1 demonstrated minimal improvement so orthoptic exercises were recommended at a gentler pace. Patient 1 returned to the clinic in October 2017 and reported that she had to discontinue her home orthoptic exercises because they continued to cause headaches and nausea. In the intervening period, she had also been diagnosed with postural orthostatic tachycardia syndrome (POTS). Review of systems revealed minimal improvement. The diagnoses of POTS and Chiari malformation raised the concern that this patient might have underlying EDS, since these problems appear to be more prevalent in patients with this syndrome [4, 5]. In the clinic, the patient demonstrated hyper-extensible elbows as well (Beighton score at least 3/9 where 4/9 is positive; note that for this patient, the complete Beighton assessment was not performed), and was referred to a geneticist for evaluation for EDS. In addition, she was provided more primitive orthoptic exercises (e.g., using a Brock string, tracking a printed labyrinth, and following a marble in a pie pan). The patient returned in January 2018 with minimal improvement in her fatigue, headaches, and dizziness, and inability to focus. She found the labyrinth and Brock string exercises uncomfortable and nausea-inducing. Review of systems was otherwise unchanged from the previous visit. A referral for evaluation for EDS by a geneticist was again provided as well as recommendations to receive accommodations so that she might better get through her daily activities. In May 2018 the patient visited the emergency room after being struck in the face next to her right eye, leading to some visual blurriness which later subsided. In October 2018, Patient 1 was assessed by a geneticist and a clinical diagnosis of Ehlers-Danlos, hypermobility type III, was made. She was recommended to establish care with a primary care physician, complete an echocardiogram, and begin visiting a physical therapist. The patient continues to be seen at the W-TBI clinic to try to address her visual symptoms. Figure 1 shows the timeline of care for this patient and all patients in this case series.

### Patient 2

Patient 2 is a 66-year-old woman who suffered two falls with secondary brief loss of consciousness in 2009 and 2014 and was diagnosed with concussion at those times. She presented to the W-TBI clinic in May 2016 after having trouble reading for many months in addition to episodes of double vision which subsided with naps since 1997. Review of systems was positive for constipation, dysautonomias (including trouble urinating and sexual dysfunction), muscle twitching and gait problems, stuttering, hyperthyroidism, chronic respiratory problems, poor memory, large changes in blood pressure, and PTSD. The patient demonstrated hypermobile joints (Beighton score 5/9) and confirmed that she was “double jointed”. Her past medical history was significant for syncope and GERD. Patient 2 was found to have convergence insufficiency, deficiency of smooth pursuit movements, diplopia, eye pain, suspected postconcussion syndrome, and dysautonomia. An MRI of the brain and orbits was ordered and the patient was provided orthoptic exercises and asked to use a walking stick for balance with re-evaluation in three months. The MRI indicated mild anterolisthesis and possible impingement upon the spinal cord. Patient 2 returned in August 2016 and reported decreased eye pain and diplopia. Review of systems was unchanged except for a new complaint of back and hip pain. A referral to a geneticist was made to consider the possibility of a hypermobility disorder due to the patient’s dysautonomia and hypermobility and the diagnosis of Ehlers Danlos syndrome, hypermobility type III, was confirmed in April 2017; notably, the Beighton score was 5/9. The patient returned to the W-TBI clinic in August 2017 and reported improved vision, although infrequent blurred vision and/or orbital discomfort still occurred. Review of systems was newly positive for GERD and possible gastroparesis, dysautonomia, pain hypersensitivity, fibromyalgia, and balance problems. The vision improvement with orthoptic therapy supported the diagnosis that the visual symptoms were secondary to concussion.

### Patient 3

Patient 3 is a 32-year-old woman who suffered a mild traumatic brain injury from a motor vehicle accident in January 2013. She was diagnosed with postconcussion syndrome and visited the W-TBI clinic in February 2014. She reported eye spasms in her right eye with pain starting the first night of the car accident, and had also been diagnosed with convergence insufficiency and accommodative insufficiency soon after that accident. Review of systems revealed fatigue, numbness and tingling in the face, right neck pain, irregular menstruation since the accident, depression since the accident, and multiple episodes of syncope since the accident. During the appointment the patient was able to follow a pendulum but became noticeably uncomfortable and reported dizziness and nausea, consistent with deficiency of smooth pursuits. Patient 3 was diagnosed with cervicogenic headaches, deficiencies of smooth pursuit movements, eyelid myokymia, postconcussion syndrome, and possible occipital neuralgia. Ehlers Danlos syndrome was suspected at this appointment based upon her demonstrating hypermobile joints (Beighton score 5/9), and a recommendation for pain management and video motion X-ray was provided to assess the damage to any ligaments in the neck and to assess for hypermobility. In February 2015, the patient was seen by a geneticist and a diagnosis of hypermobile Ehlers-Danlos syndrome was confirmed, with the video motion X-ray supporting a diagnosis of ligamentous damage and instability in the neck. In June 2015, the patient reported blurry vision with headaches at the W-TBI clinic, and in December 2015, the patient reported new episodes of double vision. The patient was seen through January 2018 at the W-TBI clinic with continued reports of severe headaches, difficult reading and concentrating, and blurred vision. The positive diagnosis of EDS led to further investigations for the patient’s ailments and several new diagnoses more commonly seen in patients with EDS were confirmed, i.e., occipital neuralgia causing cervicogenic headaches, MRI-confirmed CSF leak, and Chiari malformation.

### Patient 4

Patient 4 is a 49-year-old woman who first presented to the W-TBI clinic in February 2017 after experiencing new ophthalmic symptoms following a concussion from hitting her head on a metal beam. She had experienced three prior concussions in 1976, 1986, and 1991, and after her third concussion had developed trigeminal neuralgia which was later ameliorated with surgery. Her MRI after her fourth concussion was read as normal. However, the patient continued to experience nystagmus, dizziness, headaches, photosensitivity, and burning, dry, eyes. Furthermore, she was unable to use the computer or drive due to her visual symptoms. Review of systems at her initial appointment revealed fatigue, reflux, easy bruising, trouble urinating, dizziness and balance problems, irregular menstruation, high fluctuations in blood pressure and rate, allergies, and cognitive impairment since injury and a lifelong history of migraine. Testing convergence and smooth pursuits made the patient dizzy during the appointment, symptoms consistent with postconcussion syndrome. The patient also responded positively when asked about joint hypermobility. The history of easy bruising, migraines and labile blood pressure raised the concern that the joint hypermobility might be associated with Ehlers Danlos syndrome and the patient was referred to a geneticist. The patient was offered home orthoptic exercises and was prescribed topical cyclosporine eyedrops for her dry eyes as well as home computerized orthoptic exercises. By June 2017, the patient described being able to concentrate better and read for somewhat longer periods of time, but still had experienced carsickness and severe dry eye. In September 2018, she presented with severe pain, blurring, and feeling of a protruding left eye, and was referred for an MRI of the brain and orbits, which were read as normal. A diagnosis of hypermobile EDS was confirmed by a geneticist in November 2018.

### Patient 5

Patient 5 is a 55-year-old woman who presented to the W-TBI clinic in November 2018 after hitting her head on a piece of metal in June 2017, at which time she was diagnosed with concussion; a CT scan of the brain at the time of injury was negative. After this injury, Patient 5 found it difficult to read, reporting diplopia and nausea when attempting this visual task. Her review of systems included fatigue, constant headaches, neck pain, hypermobility, cognitive impairment, reduced memory, and depression. Her examination revealed convergence insufficiency, putative occipital neuralgia and joint hypermobility. Orthoptic exercises were prescribed and the patient was referred to a geneticist. The diagnosis of hypermobile EDS was confirmed by a geneticist in February 2019 and in March 2019 the patient returned to the W-TBI clinic reporting no improvement in her visual symptoms. The patient was referred to a pain management specialist to confirm the diagnosis of occipital neuralgia and offer treatment.

### Patient 6

Patient 6 is a 49-year-old woman who presented to the W-TBI clinic in April 2017 after being injured in a motor vehicle accident in February 2017. The patient could not recall whether she had lost consciousness following the accident. In the days following the accident, the patient started noticing nausea and headaches with reading on the computer, increased somnolence, and worsening discomfort with motion, sound, or lights. The patient had an MRI and CT following the accident which were both read as normal. At her initial appointment, the patient noted that she had enjoyed reading before the accident, but now could use a computer for only five minutes intervals prior to becoming nauseous and experiencing headache. Her past medical history was notable for breast cancer, migraines, rectal bleeding, gastric polyps, and dyspepsia. Her ROS was positive for fatigue, IBS, more headaches since the accident, increased urinary frequency, burning pain in neck and shoulders, sound sensitivity, worse bruxism since the injury, poor balance, insomnia, and cognitive impairments. The patient was diagnosed with convergence insufficiency and postconcussion syndrome and prescribed home-based orthoptic therapy. In June 2017, the patient was re-evaluated and demonstrated little improvement in her symptoms or upon examination. She did report some improvement in her fatigue, sleeping and cognition. Continued orthoptic training was recommended and that patient returned in October 2017 reporting further improvement in reading, driving, and computer use. However she stated she was not yet at her pre-morbid functioning. It was noted that the patient was hypermobile at that visit (Beighton score 4/9). In addition, she reported that the putative diagnosis of occipital neuralgia offered at previous visits was confirmed by pain management specialists. She demonstrated improved convergence insufficiency consistent with her reduced visual symptoms. The patient was referred for a genetics evaluation and in July 2018, a clinical diagnosis of Ehlers-Danlos, hypermobility type III was made. In May 2019, the patient returned to the W-TBI clinic reporting that her symptoms had “plateaued”. Her ROS was significant for fatigue, headaches, occipital neuralgia, and impaired cognition and appeared to have reached maximal medical improvement.

### Patient 7

Patient 7 is a 37-year-old female who presented to the W-TBI clinic in July 2017 after suffering a motor vehicle accident in which they did not lose consciousness. At her initial appointment, the patient reported having constant headaches since the accident, with shooting, throbbing pains in her head, photosensitivity, difficulty driving, fatigue, poor memory, and trouble reading. She reported that her headaches were more severe during the night but improved after standing upright. Her ROS was positive for fatigue, nausea, easy bruising, debilitating headaches, neck and shoulder pain, hypermobility (Beighton score 5/9), low blood pressure and dizziness when standing, and cognitive impairment. Her past medical history was significant for melanoma, breast cancer, and heavy menstrual periods. The patient was diagnosed with postconcussion syndrome with potential elevated ICP. The patient was prescribed home orthoptic exercises and recommended to sleep in the reverse Trendelenburg position for a few nights to assess for the possibility of increased ICP, since she awoke every morning with headaches and a sense of pressure in the head. Prior to re-evaluation, the patient reported that her headaches improved in the morning after sleeping in the reverse Trendelenburg position, and so an MRI with contrast was ordered to explore findings that might be consistent with elevated ICP; the MRI showed some decrease in ventricle size, possibly consistent with intracranial hypertension, but was otherwise unremarkable. The patient was offered a trial of acetazolamide in August 2017 and she reported improvement initially with this medication but then symptoms of headache and blurred vision returned. The patient returned to the W-TBI clinic in October 2017 and noted that she had worsening nausea when trying to read or use the computer and could not complete home orthoptic exercises without vomiting. Her review of systems was positive for fatigue, frequent nausea, headaches, easy bruising, hypermobility, low blood pressure and dizziness when standing up, and cognitive impairment. The patient was diagnosed with possible cervicogenic headaches and possible occipital neuralgia, convergence insufficiency, intracranial hypertension, and post-concussion migraines. She was offered brock string exercises and also referred to a pain management specialist for her neck pain. Prior to her return in January 2018, the patient received occipital nerve blocks by pain management which was unsuccessful. The patient reported inability to tolerate Brock string exercise for more than a minute at a time, with minimal improvement in reading or memory and other cognitive tasks; ROS was also unchanged. Because of the patient’s minimal improvement, history of hypermobility), and easy bruising, the patient was suspected to have a hypermobility disorder such as Ehlers Danlos. She was referred to a geneticist who confirmed the diagnosis of EDS hypermobile type III in September 2018. At a follow up in the W-TBI clinic, the patient demonstrated unimproved convergence, smooth pursuits, and stereoacuity, suggesting she had reached maximal medical improvement.

## Discussion

Brain trauma has been reported to be a triggering factor for the onset of symptoms in patients with EDS [3]. In addition, the effects of mild brain trauma on vision has been well documented [6, 7]. However, the convergence of postconcussion vision disorders and EDS has yet to be explored. In this case series, 7 patients presented with visual complaints and disorders that have been shown to be common after head injury. However, these patients are remarkable in that their visual complaints lingered for months or even years and were generally refractory to orthoptic exercises. Notably, high school and college athletes have been reported to recover from concussion within weeks [8, 9]. The fact that these patients had slower and less positive trajectories of recovery compared to most patients with concussion raised the concern that there might be an underlying problem limiting their progress. Because the review of systems taken in the W-TBI clinic for all of these patients included some degree joint hypermobility as well as a constellation of complaints often described by those suffering EDS, these patients were sent for genetic evaluations and a diagnosis of hypermobile EDS was confirmed for each. It is important to note that certain EDS signs, such as hypermobility, are not specific for EDS, and a diagnosis of EDS is usually made after thorough investigation of hypermobility using the Beighton scale, evaluation of family history, and extensive questioning investigating features of EDS such as abdominal scarring or easy bruising [2].

The possible association of lingering postconcussion symptoms and EDS suggested by this case series is important. It may be that presence of hypermobility should be discussed when treating patients with concussion. Furthermore, including the presence of absence of hypermobility as a factor in concussion databases might help determine whether EDS is indeed a risk factor for suboptimal recovery (in terms of overall progress and rate of improvement) after concussion. In addition, it could alter the management of a concussion patient, since patients with EDS who suffer trauma might express clinical findings which might not be considered during a routine evaluation after concussion, such as spinal leak or Chiari malformation. Presuming there is a correlation with poorer outcomes after concussion and EDS, it is reasonable to consider why this might be. It has been reported that reduced neck strength is a risk factor for sport-related concussion [10]. EDS patients commonly suffer both neck pain and cervical instability [11, 12]. One could argue that the necks of patients with EDS provide less protection to the head during situations leading to head injury.

In conclusion, this series of patients, summarized in Table 1, represents the first described cases where neuro-ophthalmic evaluation of patients with traumatic brain injury disclosed underlying EDS. These patients with subclinical EDS and traumatic brain injury all had slower recovery courses as well as lower levels of maximal improvement following their brain trauma. This may be attributable to the weaker connective tissues in these patients, which can create susceptibility to brain trauma but also cause slower healing. Patients presenting to a concussion clinic with persistent symptoms following traumatic events, as well as signs suggestive of EDS or joint hypermobility and personal/family history of these conditions, should undergo a thorough review of systems so that an underlying diagnosis of Ehlers-Danlos syndromes might not be overlooked.

**Table 1:** Summary of patients’ history and findings

Patient	Nature of Trauma	Pertinent Presenting Symptoms	Duration of Concussion Symptoms before EDS Diagnosis	EDS Signs	Beighton Score (> 3/9 is positive)
1	MVA	Difficulty reading and concentrating, poor visual tracking and focusing, neck pain	14 months	Chiari I malformation, POTS, hyperextensible elbows	At least 3/9*
2	Multiple falls	Difficulty reading, diplopia	11 months	Dysautonomia, hypermobile joints	5/9
3	MVA	Eye spasms, difficulty reading and focusing, neck pain	12 months	Hypermobile joints, syncope, occipital neuralgia, Chiari I malformation, CSF leak	5/9
4	Hit head on metal beam	Nystagmus, dizziness, headaches, photosensitivity, dry eye	20 months	Migraines, easy bruising, labile blood pressure	Confirmed by geneticist, Beighton score not available
5	Hit head on metal beam	Difficulty reading, diplopia, neck pain	20 months	Joint hypermobility, occipital neuralgia	Confirmed by geneticist, Beighton score not available
6	MVA	Photosensitivity, trouble reading, fatigue, headache, neck pain	15 months	Joint hypermobility, occipital neuralgia	4/9
7	MVA	Photosensitivity, difficulty reading, fatigue, headache, neck pain	14 months	Easy bruising, hypermobility, syncope, occipital neuralgia	5/9

*This patient did not have all aspects of the Beighton Test administered

## Data Availability

The datasets analyzed during the current study are available from the corresponding author on reasonable request.

## Abbreviations

EDS: Ehlers-Danlos Syndrome
POTS: Postural orthostatic tachycardia syndrome
ROS: Review of systems
W-TBI: Wilmer Traumatic Brain Injury Clinic
